# The Helpfulness of Web-Based Mental Health and Well-being Forums for Providing Peer Support for Young People: Cross-sectional Exploration

**DOI:** 10.2196/36432

**Published:** 2022-09-09

**Authors:** Emily Banwell, Terry Hanley, Santiago De Ossorno Garcia, Charlotte Mindel, Thomas Kayll, Aaron Sefi

**Affiliations:** 1 Manchester Institute of Education University of Manchester Manchester United Kingdom; 2 Kooth Plc London United Kingdom; 3 Department of Psychology University of Exeter Exeter United Kingdom

**Keywords:** adolescent mental health, peer support, web-based forums, web-based mental health

## Abstract

**Background:**

Young people are increasingly seeking out web-based support for their mental health and well-being. Peer support forums are popular with this age group, with young individuals valuing the fact that the forums are available 24/7, providing a safe and anonymous space for exploration. Currently, little systematic evaluation of the helpfulness of such forums in providing support has been conducted.

**Objective:**

This study examined the helpfulness of the support offered within web-based mental health and well-being peer support forums for young people. It specifically investigated the self-reported user ratings of helpfulness reported through the completion of a developing experience measure. The ratings will be used to consider further development of the measure and reflect upon the overall helpfulness of the forums as indicated by the reported scores.

**Methods:**

The study used routinely collected practice-based outcome data from web-based mental health forums for young people. These forums are hosted by the UK-based web-based therapy and support service, Kooth. A cross-sectional design was used to explore—using a range of inferential statistical measures—the outcomes reported by those accessing the forums using a Peer Online Community Experience Measure (POCEM). To consider the helpfulness in general, 23,443 POCEMs completed in 2020 were used. A second data set of 17,137 completed POCEMs from the same year was used to consider whether various engagement indicators had an impact upon the helpfulness rating.

**Results:**

Female users aged between 11 and 16 years predominantly completed the POCEM. This is in keeping with the majority of those using the service. In total, 74.6% (8240/11,045) of the scores on the POCEM indicated that the individuals found the posts helpful. An ANOVA indicated that male users were more likely to report obtaining intrapersonal support, whereas female users obtained interpersonal support. Furthermore, the POCEM scores reflected the internal consistency of the measure and provided an insight into the way that young people made use of the peer support resource; for instance, posts that were rated more helpful were correlated with spending longer time reading them, and the topics discussed varied throughout the day with more mental health issues being discussed later at night.

**Conclusions:**

The results seem to demonstrate that, overall, the young people involved in this study found web-based peer support helpful. They indicate that peer support can provide an important strand of care within a supportive mental health ecosystem, particularly during time periods when in-person support is typically closed. However, limitations were noted, suggesting that caution is needed when interpreting the results of this study. Although such services are incredibly well used, they have received little research attention to date. As such, further investigation into what constitutes helpful and unhelpful peer support is needed.

## Introduction

### Background

Several definitions of the term *peer support* have been proposed; yet, these can be unified in the sense that “peer support occurs between people who share similar life experiences and as a result can provide each other with reciprocal support...which professionals and/or others who have not endured the same difficult situations may not be able to” [1]. Digital web-based communities are an illustration of how peer support can be facilitated. Such communities are particularly pertinent for children and young people (CYP), who are turning to the internet with increasing frequency when seeking psychological and emotional support [2]. In 2017, 99% of the CYP aged 12 to 15 years spent approximately 21 hours a week on the web, with this figure seeming to steadily increase year on year [3,4]. Therefore, it is easy to comprehend how the practicality of web-based communities such as discussion forums is attractive to CYP [5]. This became especially true at the onset of the COVID-19 pandemic in 2020 when digital mental health support demand for CYP sharply increased [6,7]. Unlike in-person support, web-based forums are available 24/7 [8] and provide safe, comfortable platforms that are geographically unrestricted [5]. The 24/7 element of peer support forums may be especially useful for adolescents because young people’s circadian rhythms shift toward a later bedtime, with wakeful activity occurring late into the night [9,10]. Interestingly, those who experience such a shift are more likely to experience emotional and behavioral issues [11].

Even when forums are professionally led and moderated, web-based peer support is not intended to replace services provided by qualified therapists [12]. However, the fact that it is frequently available as an adjunct to other forms of mental health support [5,13] means that one can obtain additional, lower-level support that can reduce feelings of isolation [12] and normalize experiences [14]. Web-based peer support overcomes the physical obstacles that can often prevent CYP from building social relationships with others who share their experiences, allowing them to connect with peers whom they would not otherwise meet in *real life* [15]. Mental health discussion forums are particularly valued by young people who perceive themselves as lacking social skills [16]. An additional interesting finding within this body of research was that despite concerns about perpetuation of dangerous behaviors such as self-harm when young people experiencing a mental health crisis are united in a forum space, the discussion remained predominantly focused on safety and avoidance strategies [17]. This fits in with observations that people prefer sharing clinical, rather than personal and potentially identifiable information, in web-based peer support settings [18].

There are a number of challenges associated with the use of web-based forums for CYP to discuss mental health, such as ensuring user safety, building feelings of trust [5], and reducing the possibility of aggressive or unfriendly behavior that can occur as a result of user anonymity [19]. Despite these considerations, the benefits uncovered thus far in the literature mean that more in-depth examination into their use is warranted. An outline of the literature focusing on patterns of forum engagement is provided in the next paragraph.

Within forums, support can be either directive or nondirective in nature. A directive approach comprises receipt or provision of explicit advice on how to overcome an issue, and a nondirective approach involves supportively sharing experiences [14]. CYP appreciate the anonymity and privacy of peer support forums in that they find talking easier and feel less judged as a consequence [5]. This level of anonymity could play a key role in how web-based communities are built and the subsequent level of benefit that can be gained from interacting in this format. So far, findings examining this idea have been mixed. Posts on the web-based *question-and-answer* site Quora were rated as equally useful whether submitted anonymously or otherwise, and interestingly, politeness of content did not vary with anonymity [20]. Contrastingly, other findings suggested that registered forum users tended to post higher-quality posts than those who posted anonymously [21]. Ensuring that content is useful is important if web-based communities or forums are to provide a valuable and engaging method of peer support. However, to do this we must identify the factors that allow us to call a peer support forum post *helpful*. One study of interest identified several factors that contribute to forum post performance (used here as a proxy for quality) of web-based forum threaded discussions [21]. These ranged from surface-level qualities such as thread length and reply latency to less quantifiable qualities such as the perceived authority of the poster. The study suggested that factors such as thread length, or number of comments, as indicators of participative engagement can be effective determinants of thread quality; for example, they can indicate how effectively a thread answered a question or explored a topic. The number of unique users and repeat users posting on a thread are proxies for wide-reaching engagement and a desire to continue discussing a topic, respectively [21]. To our knowledge, the study by Lee et al [21] is the only one that examined forum value at such a granular level. However, as a news and current affairs forum was the platform of focus, the unique nature of peer support forums means that transferability of the study’s findings to CYP mental health forums cannot be completely assumed. There have been mixed findings regarding the question of whether factors such as number of log-ins, view counts, or page clicks, in spite of their suggested links with participation, can actually be considered true proxies of engagement. Log-in frequency was linked to engagement with web-based student learning in 1 study [22], whereas 2 similar studies found no such link [23,24]. In addition, one of these studies [24] found no link between engagement and session duration. However, all 3 studies focused on web-based learning. This again means that the findings cannot automatically be applied to digital mental health web-based forums.

It is clear from the outlined research that there are numerous positive outcomes of web-based mental health forums and communities for CYP [5,12,14]. Despite this, no empirical evidence exists that relates directly to the helpfulness of forum content that is situated within a web-based CYP peer community for mental health and well-being. As a result, this study built on recent work (Mindel, C, unpublished data, November 2021) where a Peer Online Community Experience Measure (POCEM) was developed in conjunction with a UK-based digital therapy platform for CYP. The recent preprint of the study by Mindel et al (Mindel, C, unpublished data, November 2021) detailed the POCEM’s development and piloting, as well as the beginning of the investigation of how users interacted with the measure. However, further evaluation of the POCEM within nonpilot routine service use is needed, not only in terms of how the measure itself is engaged with but also what the measure can tell us about patterns of interaction with web-based community peer support at a wider level.

### Research Questions

This study aimed to explore user-rated measurement of the helpfulness of web-based peer support forum content by investigating the following research questions:

1. What is represented in the outcomes of an experience measure (POCEM) that claims to rate the helpfulness of web-based community content?
2. What trends and commonalities exist within posts that are deemed either helpful or less helpful by CYP?

## Methods

### Design

This study used an inferential cross-sectional design [25] to identify and explore the factors that underpin the outcomes of the POCEM. Scores from routinely collected completed POCEMs were compared with several other user-level variables to explore the patterns of use stipulated by the research questions.

### Setting

The data for this study came from Kooth, which is a UK-based digital therapy and support service for CYP aged 10 to 25 years. The service provides a predominantly humanistic and idiographic approach to counseling, where users are actively involved in every step of their therapeutic journey [26]. A variety of structured or self-directed help is available through the service, with peer support community forums providing just 1 form of this available help. Service users can create and interact with forum posts on a wide variety of topics, ranging from mental health and hobbies and interests to sexuality and relationships. Worker-initiated activities and discussion threads also form part of this community to initiate discussions and encourage engagement.

No referral or joining a waiting list is needed to use Kooth, which is a free-of-charge service. However, service access is, at present, contract limited. This means that access is restricted to certain geographical regions as governed by whole-population contracts with local National Health Service trusts. Kooth can also be commissioned by other organizations, such as local authorities, for use with certain subsets of the population for whom a need for provision is recognized. Although the web-based community forums are accessible 24/7, live counselors and moderators are only active until 10 PM.

### Ethical Considerations

All data used in this study were collected from service users who had consented to their data being used for research purposes. As this was routinely collected information, the research process did not influence the data [27]. In addition, because the resulting data were anonymous and devoid of personally identifiable information, ethical review from the lead author’s institution was not required [28]. Even so, ethical principles and good practice guidelines for managing information of this nature were followed [29].

### Measures and Data Sets

#### Overview of the POCEM

The POCEM is a 3-step web-based experience measure designed for users to rate content within the Kooth community. It aims to measure satisfaction with, and quality of, web-based community resources in relation to how well they met the expectations of the service user completing the measure. Any time a service user engages with a community item such as a forum post or an activity, they are, as a first step, asked how helpful they found it by rating it on a Likert scale (-2: No; -1: Not really; 0: Not sure; 1: A bit; 2: Loads). The second and third steps are explained in the Data Set 1 section.

#### Data Set 1

Two data sets were used to investigate the research aims. For the first aim—to examine the user-rated measurement of helpfulness and experiences gained from community forum interaction—a cross-sectional extraction of every POCEM completed in 2020 was performed. This data set of 23,443 POCEMs was completed by 11,045 unique users. Each user completed a mean of 2.12 (SD 2.64) POCEMs. This data extraction captured the second and third steps of the 3-step measure. In the second step, after assigning a numerical score to a post, the service user was prompted to indicate a reason for their rating, choosing from 4 options to reflect the nature of their experience. This was optional, and their Likert rating choice was recorded even if they chose not to engage further. In addition, if the user selected *not sure*, the interaction with the measure would end at this point. The 4 experience domain options to choose from are based on 4 high-level outcomes [30] that were devised from the different types of support that are commonly sought (refer to the *High-level support represented by each domain* column in Table 1). Third and last, users were then given the option to provide more information about their experience by selecting from a range of statements that correspond to the domain that they had selected. These additional statements (refer to the *Additional selectable statements* column in Table 1) represent indicators of outcomes of a positive community experience, and users could choose one or more of these. This data set also captured the demographics of age, gender, and ethnicity of users completing the measure, as well as the topic category to which the POCEM-rated content related; for example, *mental health*, *bullying*, or *friends*.

**Table 1:** The selectable domains and their corresponding statements that are available in the Peer Online Community Experience Measure and the high-level support outcomes that they represent.

Service user–experience domains	Additional selectable statements under each domain	High-level support represented by each domain
“I want information about something important to me” (Important to me)	I got information that helped me learn about myself The information I received today was helpful to my problem I now know what I need to do to feel better I learned something new today I now know that others have the same experiences as me	Informational intrapersonal support
“I want to learn some skills to try with other people” (Learn skills)	I have learned how to express myself I have learned enough to make a positive change I have developed skills to open up more I now have knowledge and skills to help others I have learned how to support others	Informational interpersonal support
“I want to explore more about how I relate to other people” (Relate to others)	I feel safe in the Kooth community I felt connected to someone I feel that I’m just as valuable as others I know who to ask for help I feel motivated to give advice to others It feels good not to be judged	Emotional interpersonal support
“I want to understand myself more” (Understand myself)	I felt accepted I now feel more hopeful I now feel able to ask for support outside of Kooth My problems now feel more manageable I am now able to find solutions to my problems I now want to make changes in my life I no longer feel alone	Emotional intrapersonal support

#### Data Set 2

The second data set, a subset of data set 1, comprised 17,137 POCEMs completed by 10,612 unique users. In this data set, an average of 1.62 (SD 1.89) POCEMs were completed per user. It was used to explore the nuances and commonalities that exist among posts that were rated a certain way, as per the second research aim. This data set was a cross-sectional data extraction of those POCEMs completed within 1 month of their corresponding community forum post being submitted. As a clarifying example, a POCEM that was completed on April 21 that related to a forum post submitted on March 22 would be included in this extraction; however, a POCEM completed on April 23 would not be included. This limit was set because engagement with posts tends to become sparse when they are >1 month old. As in the first data set, a measurement score as well as the category topic of the community forum post were captured. The number of comments on, and views of, a post at the time of each POCEM completion were ascertained too. Owing to the interface of Kooth community forums, these features related to what a user can see when they enter the forum environment. The amount of time each user spent interacting with the post (in minutes) before they submitted the measurement scores, the average score of already submitted POCEMs, and the age of each post (in days) when each POCEM was completed were also recorded. In the Kooth web-based community, forum posts do not move to the top of the page when a new comment is submitted. If the age of posts is linked to POCEM completion likelihood and score, it could provide useful insight into how users search for, and engage with, posts.

### Data Analysis

#### Data Set 1

Frequency tables were produced to explore the demographics of those who completed POCEMs, the distribution of scores, and the topics to which the content related. The split of gender and ages across the 4 experience domains (Table 1) were explored with 1-way Welch ANOVAs, which were chosen because the data violated the *homogeneity of variance* assumption [31]. For these tests, age was divided into ≤13 years and ≥14 years. This boundary was chosen owing to the developmental transition into adolescence that is commonly found to occur around this age and because of the finding that the peak age of onset of any diagnosable mental health issue is 14.5 years [32]. Consequently, these 2 age groups may approach peer support in different ways. We chose not to further split this exploration by topic category because these varied so drastically in representation; for example, 26.49% (6210/23,443) of the POCEMs that were completed related to the mental health topic, but only 0.04% (10/23,443) of the POCEMs that were completed related to the independence topic.”

Heat maps were produced to explore which pairs of corresponding statements (refer to the *Additional selectable statements* column in Table 1) were the most frequently selected together for each domain. Heat maps are a form of data visualization where tiles in a matrix are shaded based on the combined value of the corresponding axes. They are often shaded from red (low frequency or value) to green (high frequency or value) [33]. They were incorporated into this study to explore any potential patterns of consistencies or contrasts that exist in the service user experience. This was carried out by using Microsoft Excel’s COUNTIF function to create dummy variables that detected the presence of each statement across each individual POCEM, then using the crosstab function of SPSS software (version 25.0; IBM Corp) to obtain counts of pairs to be inputted into the heat maps.

#### Data Set 2

A simple correlation matrix explored the potential linear relationships among the various qualities of a community post at the time of POCEM completion. Next, a new variable was computed to split the data based on the time of day that each POCEM was completed to investigate how CYP use their free time outside of education or work to take care of their mental health and what patterns of completion followed after 10 PM when live counselors are offline. Using this variable, 1-way Welch ANOVAs investigated whether POCEM scores, time spent reading a post within the web-based community forum, and the post’s age at POCEM completion varied depending on the time of day. Frequency tables examined trends relating to the topics interacted with at different times of day, with further ANOVAs used to explore points of interest by isolating topic categories through the computation of dummy variables.

## Results

In the following sections, we detail the findings of the investigations outlined in the Methods section. Please note that where dummy variables were inputted into significance tests, means are not provided because they would represent transformations rather than meaningful reflections of the original data.

### Data Set 1

In total, 75.3% (17,653/23,443) of the POCEMs in this data set were completed by female users and 18.9% (4431/23,443) by male users, whereas 3.7% (867/23,443) and 2.1% (492/23,443) were completed by respondents who identified as gender fluid and agender, respectively. In total, 91.2% (21,380/23,443) of the measures were completed by users aged between 11 and 16 years, which corresponds with secondary school age in the United Kingdom. Of the 11,045 users, 4628 (41.9%) were aged 12 or 13 years, and only 376 (3.4%) were aged >18 years. In terms of ethnicity, 79.79% (8813/11,045) of the sample identified as White (White British, White Irish, or “other White background”), which falls below the 86% reported in the 2011 England and Wales census [34].

In terms of helpfulness scores, 74.6% (8240/11,045) of the users selected *1* or *2*, indicating that they found the community forum post helpful, whereas 13.5% (1491/11,045) were unsure, and 12% (1325/11,045) found the post unhelpful. POCEMs were completed on posts relating to 16 topics. In total, 26.49% (6210/23,443) of the POCEMs were completed for mental health–related posts, with the next most common being sex and relationships at 13.63% (3196/23,443).

Significant gender differences existed in the likelihood of choosing the domains *important to me* (*F*_3,1037.5_=2.845; *P*=.04) and *understand myself* (*F*_3,1041.54_=2.834; *P*=.04), with more male users than female users choosing these as reflective of their community experience. A difference was also found for the domain *relate to others* (*F*_3,1040.28_=4.965; *P*=.002), but conversely, more female users than male users chose this. No gender differences existed in selecting the *learn skills* domain, and those identifying as gender nonconforming did not differ significantly from male users or female users in any of their choices. CYP aged ≥14 years were significantly more likely than those aged ≤13 years to choose *relate to others* (*F*_1,13555.74_=82.66; *P*<.001). Those aged ≤13 years were more likely than those aged ≥14 years to select *important to me* (*F*_1,13999.15_=12.05; *P*=.001), *learn skills* (*F*_1,14207.18_=34.78; *P*<.001), and *understand myself* (*F*_1,13741.59_=15.85; *P*<.001).

**Figure 1:**
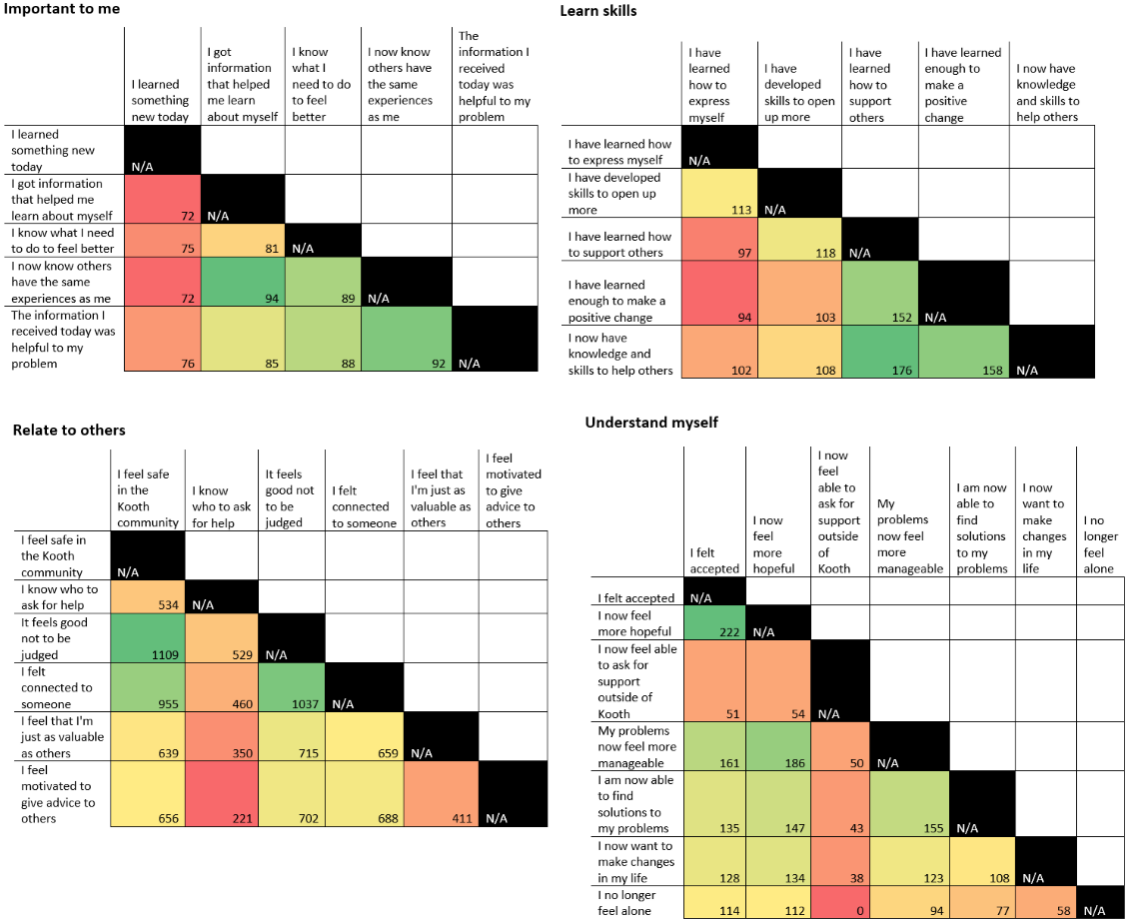
Heat maps showing the concentration of pairs of selectable statements. Those selected together with the highest frequency are dark green, with red indicating the lowest frequency. N/A: not applicable.

Figure 1 shows heat maps that show the concentration of the pairs of additional selectable statements that were chosen together under each service user–experience domain. The pairs that were selected together the most frequently are in darker green, with the red squares representing pairs chosen together less often. The heat maps contain data only from those POCEMs where at least two statements were selected (3241/23,443, 13.83%).

### Data Set 2

Negligible yet significant correlations were found between the POCEM score and age of the post at time of measure completion (*r*_17,135_=0.03; *P*<.001) and between the POCEM score and time spent on the post (*r*_17,135_=0.08; *P*<.001).

Significant correlations were also found between the age of the post and the following variables: time spent on the post (*r*_17,135_=0.16; *P*<.001), average score of previously submitted POCEMs on the post (*r*_17,135_=0.42; *P*<.001), and total number of submitted measures on the post (*r*_17,135_=0.40; *P*<.001).

The amount of time in minutes a user spent reading or otherwise interacting with a post before completing a POCEM was significantly correlated with the number of views that the post had at the time of POCEM completion (*r*_17,135_=0.31; *P*<.001), number of comments at the time of POCEM completion (*r*_17,135_=0.49; *P*<.001), average score of previously submitted POCEMs (*r*_17,135_=0.28; *P*<.001), and total number of submitted POCEMs on the post (*r*_17,135_=0.37; *P*<.001).

When the POCEMs were broken down per the time of day in which they were completed, 48.9% (8380/17,137) were completed between 4 PM and 10 PM (*evening* and *night*), with 39.7% (6803/17,137) completed between 6 AM and 4 PM (*morning* and *afternoon*). In total, 11.4% (1954/17,137) of the POCEMs in the sample were completed between 10 PM and 6 AM (*late night*).

The POCEM scores did not vary depending on time of day (*F*_4,7248.25_=0.841; *P*=.499). However, significant main effects of post age at time of POCEM completion (*F*_4,7166.89_=31.14; *P*<.001) and time spent engaging with a post before POCEM completion (*F*_4,7445.44_=3.52; *P*=.007) were found. Games-Howell post hoc tests revealed several significant differences (Tables 2 and 3).

**Figure 2:**
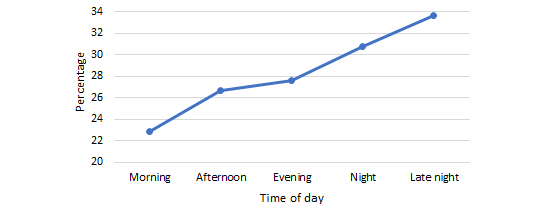
Percentage of total Peer Online Community Experience Measures completed that related to a mental health–themed post at different times of day.

**Figure 3:**
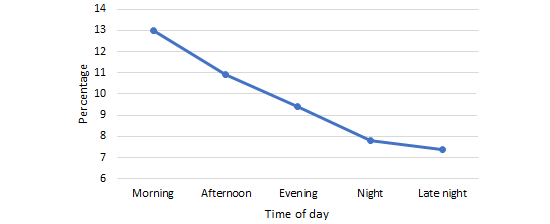
Percentage of total Peer Online Community Experience Measures completed that related to a hobbies- or interests-themed post at different times of day.

When examining the topics of forum posts for which POCEMs were completed at different times of day, the most popular topics were checked for apparent patterns. In the *morning*, between 6 AM and noon, 22.9% (3924/17,137) of the POCEMs were related to a mental health–themed forum post. This percentage increased as the day progressed, reaching 33.6% (5758/17,137) at *late night* (Figure 2). However, hobbies or interests followed an opposite pattern. In the *morning*, 13% (2228/17,137) of the POCEMs were related to this topic, but by *late night*, this fell to 7.4% (1268/17,137; Figure 3).

Exploring these phenomena, measure completion for mental health–related posts significantly varied depending on time of day (*F*_4,7167.85_=21.95; *P*<.001). Games-Howell post hoc tests showed that all pairwise comparisons were significant, besides night and late night (*P*=.22) and afternoon and evening (*P*=.87).

POCEM completion for hobbies- or interests-related posts also significantly varied depending on time of day (*F*_4,7292.65_=16.26; *P*<.001). All pairwise comparisons were significant, besides night and late night (*P*=.99), afternoon and evening (*P*=.11), evening and night (*P*=.16), and evening and late night (*P*=.09).

**Table 2:** Games-Howell pairwise comparisons among times of day in terms of forum post age (in days) at the time of Peer Online Community Experience Measure completion.

Time of day (mean post age in days) and pairwise comparison			*P* value
**Late night (mean 4.03, SD 4.35)**			
	Morning	.003^a^	
	Afternoon	.01^a^	
	Evening	.12	
	Night	.003^a^	
**Morning (mean 4.48, SD 4.21)**			
	Late night	.003^a^	
	Afternoon	.99	
	Evening	<.001^a^	
	Night	<.001^a^	
**Afternoon (mean 4.43. SD 4.40)**			
	Late night	.01^a^	
	Morning	.99	
	Evening	<.001^a^	
	Night	<.001^a^	
**Evening (mean 3.76, SD 4.07)**			
	Late night	.12	
	Morning	<.001^a^	
	Afternoon	<.001^a^	
	Night	.32	
**Night (mean 3.58, SD 4.04)**			
	Late night	.003^a^	
	Morning	<.001^a^	
	Afternoon	<.001^a^	
	Evening	.32	

^a^Values that met the significance threshold (*P*<.05).

**Table 3:** Games-Howell pairwise comparisons among times of day in terms of time spent engaging with a post (in minutes) before Peer Online Community Experience Measure completion.

Time of day (mean time spent engaging with post in minutes) and pairwise comparison			*P* value
**Late night (mean 0.82, SD 0.62)**			
	Morning	.02^a^	
	Afternoon	.34	
	Evening	.03^a^	
	Night	.89	
**Morning (mean 0.88, SD 0.64)**			
	Late night	.02^a^	
	Afternoon	.62	
	Evening	.99	
	Night	.14	
**Afternoon (mean 0.86, SD 0.67)**			
	Late night	.34	
	Morning	.62	
	Evening	.78	
	Night	.87	
**Evening (mean 0.88, SD 0.83)**			
	Late night	.03^a^	
	Morning	.99	
	Afternoon	.78	
	Night	.23	
**Night (mean 0.84, SD 0.66)**			
	Late night	.89	
	Morning	.14	
	Afternoon	.87	
	Evening	.23	

^a^Values that met the significance threshold (*P*<.05).

## Discussion

### Overview

Web-based peer support, especially that pertaining to CYP mental health, has received very little direct research attention thus far despite its extensive use by this demographic. To close this gap, this study builds on a recent measure development paper (Mindel, C, unpublished data, November 2021) by providing novel insight into how CYP interact with a web-based community measure designed to rate the helpfulness of peer support forum content. From the information provided in the POCEMs, we were able to investigate the commonalities underpinning forum posts and web-based community content that were rated in certain ways.

### Outcomes of the POCEM

#### Demographics

In the first data set, user-rated measurements of helpfulness were examined. In total, 41.9% (9823/23,443) of the POCEMs within this extraction were completed by users aged 12 or 13 years. These ages are characterized by the transition from childhood into adolescence. Many social, educational, and biological changes affect a young person’s life at this time [35]. In addition, approximately half of mental health conditions are commonly believed to begin before the age of 14 years [36], and the peak age of onset of any diagnosable mental health difficulty is 14.5 years [32]. This age clearly represents a crucial period for which attention to supportive prevention should be paramount. Ensuring that services, including those that are web based, provide targeted support for the specific issues faced by this age group may help with this. It is also worth noting that the low percentage of users aged >18 years in the sample (376/11,045, 3.4%) could be explained by this age group’s eligibility for Kooth’s adult-specific digital therapy platform, Qwell.

We also found that considerably more POCEMs were completed by female users (17,653/23,443, 75.3%) than male users (4431/23,443, 18.9%). Data from the Millennium Cohort Study found that emotional mental health symptoms increased from 12% to 18% for girls aged between 11 and 14 years, although prevalence for boys stayed the same [35]. Although this higher susceptibility might have partial responsibility for the gender difference in use that we found, the magnitude of the difference could suggest that other factors are also in play. Gender differences in help-seeking behavior are well documented, with women more likely to visit their general practitioner for any reason [37], including mental health concerns [38]. This difference unsurprisingly extends to adolescents, with the stigma and gender stereotypes surrounding masculinity and strength [39,40] influencing mental health help–seeking likelihood. Interestingly, although female adolescents also tend to be better at identifying psychological problems and possess greater knowledge of available help, education level is also a significant predictor of these factors [41]. Although our finding contributes to the idea that targeted mental health awareness for men and boys is undeniably required, an overall increase in mental health discussion and signposting could help to bridge any educational or socioeconomic differences that exist in terms of mental health literacy and resultant access to support.

#### User-Reported Experiences

More male users than female users chose the domains *important to me* and *understand myself* as reflective of the experience they had with the community post and content, and more female users than male users chose *relate to others*. This suggests that male users may approach web-based community forums with a desire for intrapersonal advice, with female users more likely to seek interpersonal support. Although little research evidence exists to corroborate these findings, they could tentatively match patterns of help-seeking strategies described in a recent study [39], where male participants preferred to adopt self-reliance when dealing with psychological problems. By contrast, female participants tended to have more confidence in mental health professionals. However, the same study found that male participants, more so than female participants, find their friends more helpful under such circumstances [39]; this is a finding that is not concordant with ours. Interestingly, although female adolescents may desire stronger connection from their friendships, perhaps explaining their increased likelihood of choosing *relate to others*, friendships are valued as equally important by both [42]. In terms of age, we found that older CYP (≥14 years) who completed the POCEMs selected *relate to others* more readily than those aged ≤13 years, with the latter group more likely to choose all 3 of the other experience domains. The association between peer support and well-being becomes stronger for CYP with age [43], which could explain this finding. Friendship, in particular dyadic friendships and the unique social nuances and problems that arise in them, become increasingly salient during early adolescence [44,45].

Figure 1 shows the frequency with which pairs of additional selectable statements were chosen together when users elaborated on the experiences that they had with community content forum posts. We will now discuss some of the key trends that can be inferred from these heat maps. An overall observed pattern was that the statements selected together tended to be conceptually similar; for example, under the *learn skills* domain, *I now have knowledge and skills to help others* and *I have learned how to support others* were chosen as the most frequent pair, both of which are almost identical in terms of the experience reported. Conversely, those least frequently selected together were often clear opposites; for example, under *relate to others*, the items *I know who to ask for help* and *I feel motivated to give advice to others* refer to receiving and providing advice, respectively. Therefore, it is unsurprising that users do not report getting both experiences from a single forum post. Looking more closely at specific trends within each heat map in turn, under *important to me*, the statement *I learned something new today* was the least selected overall, with *I now know that others have the same experiences as me* combined with both *I got information that helped me learn about myself* and *the information I received today was helpful to my problem* representing the most frequent pairings. The latter 2 statements can be viewed as broad, unspecific positive experiences that can easily be combined with others. This point about statement specificity is also relevant for the domain *learn skills*, where the least frequently selected statements *I have learned how to express myself* and *I have developed skills to open up more* can be viewed as less broad than other statements in the domain. They also seemingly relate to personal gain, whereas the others relate more closely to gaining skills to help others. Under *relate to others*, the items *I feel safe in the Kooth community*, *it feels good not to be judged*, and *I felt connected to someone* were commonly selected together, seemingly encompassing a sense of comfort and security. Finally, in *understand myself*, the most frequent pairings, of which *I felt accepted* and *I now feel more hopeful* is 1 example, seem to relate conceptually to positivity and a better appraisal of the users’ situation. *I now feel able to ask for support outside of Kooth* was the least commonly selected outcome statement in this domain. It stands out as distinct and not truly relating to the *understand myself* domain, perhaps explaining why it was not chosen if self-understanding was the key overarching experience gained.

### Trends of Forum Post Helpfulness

#### Qualities of Forum Posts

In the second data set, significant correlational relationships of varying strengths were found among the qualities of a post at each instance of measure completion. Posts with higher POCEM scores were older, and users spent longer time reading them. Although these correlational relationships were negligible, post age and time spent interacting were key factors of interest, and more POCEMs were also completed on older and longer-read posts. As mentioned earlier, within Kooth’s forums, posts do not move to the top of a page when a new comment is posted. In addition, the only way to attempt to locate relevant posts is by selecting a topic category. Accessing and completing the measure for an older post therefore implies a targeted search for that content: the user has likely scrolled through several pages and selected a topic to find information pertaining to their issue. They have likely logged in and spent time engaging with the community because of a specific want or need. Once they find the relevant information, it is likely to be viewed more favorably because of this specific need resolution. We also found that older posts were engaged with for longer before POCEM completion, which further strengthens this idea of targeted searches being more useful—more time is spent reading and absorbing the information within, leading to increased likelihood of POCEM completion and higher rating. Posts interacted with for longer before POCEM completion also had more comments and views. Comments and views are the only 2 engagement figures visible to forum users; therefore, high numbers may elicit a curiosity that results in longer reading time. These relationships all suggest that a more user-friendly search function would be beneficial. Older posts are evidently scrolled through, interacted with, and deemed useful, and the findings suggest that CYP are using the forums to search for information and to seek help in a targeted fashion, in addition to general browsing and interacting. A search-term function, in addition to a facility to sort posts by factors such as *most popular*, would prove useful for users, as well as maximize the research potential of the POCEM itself. In summary, these links among factors such as time spent, post age, and POCEM completion do imply that feedback mechanisms such as the POCEM can be seen not only as proxies of engagement but also as helpful tools for building understanding of web-based forum behavior. More interdisciplinary research of this kind could help us to understand whether engagement and interaction behavior differ depending upon the nature of the web-based system used, be it educational, support seeking, or any other kind. This is due to the fact that our findings do not completely align with studies that looked at engagement in different types of platforms [23,24].

#### Time of Day

Looking at POCEM completion across times of day in the web-based community proved insightful, particularly in terms of how CYP choose to spend their time outside of school or work—their *leisure* time. Of the 17,137 POCEMs in data set 2, 10,334 (60.3%) were completed outside of standard working hours (between 4 PM and 6 AM), allowing us to make the broad claim that CYP are actively choosing to engage with community peer support during their free time. Figures 2 and 3 show that POCEMs were completed for significantly older posts in the morning and afternoon than in the evening, night, and late night. Although this may tenuously suggest that users are searching through older posts during the day, it may simply reflect the fact that higher overall engagement after 4 PM likely includes creation of more new posts at this time. Therefore, if a user logs in between 6 AM and 4 PM, the posts they interact with are likely to be slightly older. This brings to light a limitation that should be considered with this main effect as well as the aforementioned correlational findings: to infer user motivations behind completing an experience measure for older posts, we would need to know the average age and rank of the posts that appear on the first page of the forum when users log in. Although some users in the sample did complete POCEMs for posts that were up to 30 days old, the mean age of a post at POCEM completion was 4.04 (SD 4.21) days. Therefore, interacting with a slightly older post does not necessarily suggest, in all instances, a targeted search. More information about post rate and subsequent first-page turnover is necessary to substantiate this claim. Users also spent longer time engaging with posts before completing the measures in the morning and evening than late at night. Any explanations for this, such as a user having more comments to catch up on in the morning and evening or that more targeted searches late at night perhaps equate to a quicker decision regarding a post’s helpfulness, would be purely speculative.

Arguably, the most interesting findings concerning time of day appeared when we examined the topics of posts that were rated at different times of day. As shown in Figure 2, of the posts relating to mental health, 33.61% (656/1952) were completed late at night, whereas 22.87% (739/3231) were completed in the morning. This suggests that users logging into the web-based service at night and in the very early hours are doing so to seek mental health support. This idea is also strengthened when we look at hobbies or interests, a contrasting topic that is more conversational and less advice centric. Significantly more measures on this topic were completed in the morning than at night (Figure 3). How, then, can we explain this seemingly increased focus on mental health at night? Why are those who are awake at night interacting with mental health topics? Insomnia has frequently been linked to an increased likelihood of mental health problems, including in adolescents [46,47]. In addition to insomnia, *eveningness*, often colloquially known as being a *night owl*, is also independently associated with psychopathology in adolescents [11]; however, only insomnia increased the risk of suicide ideation [48]. CYP commonly shift to a pattern of *eveningness* as they enter adolescence, and societal pressures such as school start times do not accommodate this [9,10], perhaps explaining why these CYP have adverse mental health outcomes even in the absence of disordered sleep. In terms of support seeking, patients seeking psychiatric support outside of daytime care hours tend to present with mental health issues of greater severity and complexity [49,50]. The direction of the relationship between poor mental health and being awake at night, either through insomnia or individual differences in sleep patterns, is unclear. However, what is apparent is that users who are awake while most of their peers are asleep are likely to be those with the most complex mental health issues, which lack of sleep may exacerbate their tendency to ruminate upon at this time [51]. The key suggestion with regard to this finding, although more research is needed to further pinpoint patterns of user activity *after hours*, is to ensure that appropriate provision is in place to support CYP who seek mental health support at this time of day; for example, making sure that round-the-clock crisis helplines such as Nightline (a support line for students in the United Kingdom), The Samaritans, or Childline are especially visible to users after 10 PM when live support is unavailable, with users encouraged to call the helplines if they feel that they require live support urgently.

### Strengths, Limitations, and Future Directions

Although several useful insights were gained from this research, they must be viewed in the context of the following limitations. The first of these relates to the study’s cross-sectional design. The large data set of 23,443 POCEM completions was an asset of the study, given the statistical power this offered our analyses. However, examining trends over time by means of a longitudinal design would allow us to explore how forum use and user experiences fluctuate over time. This would be of particular interest during times of widespread crisis, such as the COVID-19 pandemic. This would also allow examination of how long-term forum users engage. Linked to this idea, we mentioned in the Methods section that the 23,443 completed POCEMs came from 11,045 unique users. Given that the study’s aims primarily focused on post helpfulness rather than repeated patterns by users, our analyses did not account for the fact that many users completed the measure multiple times. We suggest that each POCEM represents a valid and distinct user experience. However, future longitudinal research could investigate user intensity and patterns of response in 1-time users compared with repeat users. This could be to identify whether helpfulness ratings differ and whether users tend to report the same or different experience domains whenever they interact with the POCEM.

It is also wise to acknowledge the limitations that exist with the finding that 74.6% (8240/11,045) of the POCEM scores were positive. Ratings of satisfaction tend to be completed more frequently by those at opposite ends of the experience spectrum. This extremity bias [52] means that people are very keen to report an excellent or a terrible experience but are less inclined to report an average or uninteresting one. Although these biases are most often reported in consumer research, our finding could easily be explained by this phenomenon, with forum posts perhaps being subject to an even starker imbalance—one that echoes the positively skewed J-shaped distribution curve often reported in web-based product reviews [53]. A forum post, as content nested within the web-based peer community, is likely to either be *very* helpful to a CYP or unhelpful in that it is viewed as either boring or irrelevant. Practitioner moderation means that content in the community forum is unlikely to be actively harmful or damaging, meaning that the only *extreme* experience a CYP can have is a positive one. Therefore, finding a post mediocre is less likely to result in completion of the measure. Encouraging review submission may result in a more even distribution of ratings [52]. Although care must be taken not to pressure users or create an interface that is an annoyance to interact with, increasing the visibility of the measure inside of the peer community forum may help reduce this bias. This limitation, realistically, applies to all findings in this study when we think about *who* completes a web-based community measure such as this one and *why*. Social desirability and acquiescence biases may also be at play, given the positive skew and given that these bias effects are often seen in digital scale measures [54,55]. However, these effects were raised and addressed in the piloting stage of POCEM development (Mindel, C, unpublished data, November 2021), and consequently, a reassuring message regarding anonymity and confidentiality was added to the measure before the present data were collected. This has hopefully ameliorated any worries that users may have about their responses being linked back to them. It should also be noted that time spent completing the POCEM was included in the recording of time spent interacting with a forum post in data set 2. This time should certainly be separated in future research into the POCEM because it could then be ascertained whether users were reading and engaging with the questions the POCEM asks or whether they were simply clicking random answers. Comparing POCEM completion time with time spent engaging with the related forum post would also prove insightful.

There are also several methodological constraints relating to the use of heat maps (Figure 1). Although heat maps are attractive forms of data visualization, it is important to bear in mind that they are not, and should not be treated as, complete data analyses in their own right [56]. The findings relating to Figure 1 should therefore be treated as *patterns* of engagement rather than true differences owing to the lack of significance testing in heat maps. Along these lines, the colors used when shading heat maps can make differences appear large, even if the gap between the biggest and smallest value is very small. If they are to be used as analytic tools, measures of central tendency, at the very least, should be included. Heat maps were included in this study for identification of trends and patterns rather than to identify definitive differences. Therefore, provided that these limitations are considered when interpreting the aforementioned findings, they remain a useful way to visualize this element of our data.

### Conclusions

To summarize this research, this study used a large data set to explore the use of a user-rated helpfulness measure in a CYP mental health peer support forum context. The results seem to indicate that the young service users involved in this study found web-based peer support helpful and that the POCEM itself was engaged with well. This suggests that peer support can provide an important strand of care within a supportive mental health ecosystem, particularly during time periods when in-person support is typically closed. This latter finding stresses the importance of vigilant support provision at these times. Measures were most often completed by secondary school–aged CYP and by many more female users than male users. The age of a post and the time spent engaging with a post before POCEM completion were found to be factors of interest, providing insight into the search habits of users. These factors denote key areas where peer support forums can be made more intuitive and user friendly. Caution is advised when interpreting the results of this study. Although such services are popular, and indeed useful, they have received little research attention to date. As such, further investigation into the nature of helpful and unhelpful peer support is warranted, as well as increased focus on how it should be incorporated into comprehensive systems of digital mental health support.

## Abbreviations

CYP: children and young people
POCEM: Peer Online Community Experience Measure
